# Sustained NF-κB-STAT3 signaling promotes resistance to Smac mimetics in Glioma stem-like cells but creates a vulnerability to EZH2 inhibition

**DOI:** 10.1038/s41420-019-0155-9

**Published:** 2019-03-04

**Authors:** Cintia Carla da Hora, Kelsey Pinkham, Litia Carvalho, Max Zinter, Elie Tabet, Ichiro Nakano, Bakhos A. Tannous, Christian E. Badr

**Affiliations:** 1 Department of Neurology, Massachusetts General Hospital, Boston, MA USA; 2 Neuroscience Program, Harvard Medical School, Boston, MA USA; 3 Experimental Therapeutics and Molecular Imaging Laboratory, Charlestown, MA USA; 4 Department of Neurosurgery and Comprehensive Cancer Center, University of Alabama at Birmingham, Birmingham, AL USA

**Keywords:** Cancer stem cells, CNS cancer

## Abstract

Glioblastoma is an incurable and highly aggressive brain tumor. Understanding therapeutic resistance and survival mechanisms driving this tumor type is key to finding effective therapies. Smac mimetics (SM) emerged as attractive cancer therapeutics particularly for tumor populations that are highly resistant to conventional apoptosis-inducing therapies. We evaluated the therapeutic efficacy of SM on Glioma stem-like cells (GSCs) and showed that this family of compounds stimulates an adaptive response triggered by TNFα. Increased expression of TNFα results in a prolonged and sustained activation of NF-κB and STAT3 signaling thus activating several tumor cell resistance mechanisms in GSCs. We show that STAT3 activation is contingent on EZH2 activation and uncover a synergistic lethality between SM and EZH2 inhibitors. Therapeutic inhibition of EZH2 impaired the viability of SM-treated GSCs. Our study outlines the molecular underpinnings of SM resistance in glioblastoma and provides mechanistic insight to overcome this resistance and increase therapeutic efficacy.

## Introduction

Glioblastoma (GBM) is the most malignant and most common form of primary central nervous system tumors with high mortality and resistance to therapy. Within GBM tumors, lies subpopulation of cells with stem-like properties, referred to as glioma stem-like cells (GSCs). Owing to their self-renewal properties, plasticity and inherent resistant to therapy, these cells promote GBM progression, invasion, and recurrence^1^.

The activation of transcription factors, induced by extrinsic factors such as the tumor microenvironment or therapeutic stimuli, can transform the genetic landscape of GBM tumors, which can be reflected by an increased mesenchymal signature^2^. These changes are commonly associated with invasion, increased self-renewal, and proliferation, as well as therapeutic resistance^3^. Pro-inflammatory cytokines, such as TNFα and IL-6 secreted in the tumor microenvironment by immune cells or tumor cells, can drive tumor plasticity and increase cancer stem cell maintenance, as observed in numerous malignancies including GBM^2^. In GBM for instance, Nuclear Factor-κB (NF-κB) activation, which correlates with poor patient prognosis, promotes mesenchymal differentiation and therapeutic resistance as shown by several groups including our own^2,4^. Aberrant NF-κB signaling promotes the release of pro-inflammatory cytokines and the activation of several oncogenic transcription factors including Signal transducer and activator of transcription (STAT3)^2^. STAT3 signaling is essential to maintain self-renewal and proliferation of GSCs^5^, and promotes a mesenchymal transition in GBM^2,6^.

IAPs (Inhibitors of apoptosis) represent a family of proteins that primarily act as endogenous inhibitors of caspases thus preventing apoptotic cell death^7^. Several members of the IAPs family including cellular IAP2 (cIAP2) have increased expression in Gliomas, which correlates with poor prognosis and can potentially favor therapeutic resistance in GBM^8^. IAPs antagonists, commonly known as SMAC Mimetics (SM), have been developed to counteract apoptotic resistance in cancer cells, and several are being evaluated in clinical trials^9^. SM primarily induce the autoubiquitination and degradation of IAP1 and IAP2, resulting in activation of NF-κB, increased expression of its target cytokine TNFα and subsequent TNFα-mediated cell death^10^. Because of this ability to stimulate cytokine release, SM have emerged as potent adjuvants to GBM immunotherapy^11,12^.

Due to their inherent resistance of GSCs to apoptotic stimuli and given the relevance of IAPs and SM in GBM, we sought to evaluate the therapeutic efficacy of SM on this cancer stem cell population. In this study, we provide a comprehensive overview of the molecular mechanisms that support or promote therapeutic resistance to SM in GSCs.

## Results

### GSCs are inherently resistant to SM

**Fig. 1:**
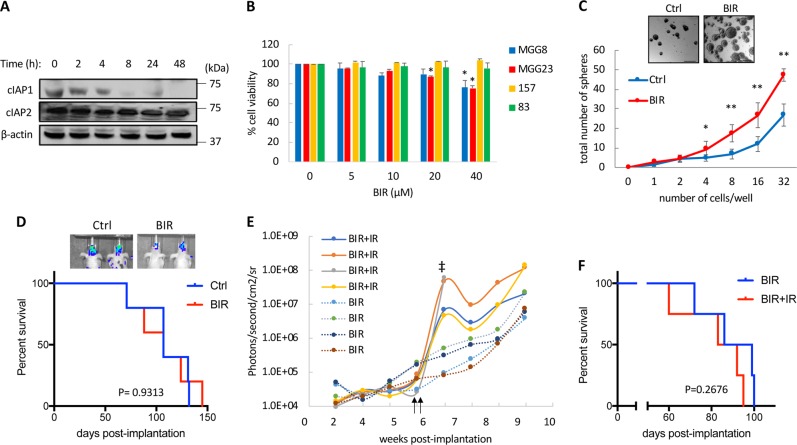
SM fail to achieve therapeutic efficacy in GSCs. **a** Western blot analysis of cIAP1 and cIAP2 expression at different time points in MGG23 GSCs treated with BIR (10 µM). **b** Four GSCs were treated with BIR at the indicated doses and cell viability was assayed after 7 days. Data presented as average +/− SD where the untreated control is set at 100%. **c** Sphere formation assay in 157 GSCs treated with BIR (2 µM) for 11 days. Micrographs of neurospheres are shown in the upper panel. Scale bar, 100 µm. **d** BT07-Fluc GSCs were treated with BIR (2 µM) every 3 days for 20 days before brain implantation in mice (*n* = 5/group). Kaplan–Meier curves showing median survival in both groups. (*P* values, two-sided log-rank test). The upper panel shows representative bioluminescence images of mice from each group at week six post-implantation. **e** Mice bearing MGG6-Fluc cells treated ex-vivo with BIR prior to implantation (*n* = 4/group). Tumor growth was monitored overtime using Fluc imaging. Vertical arrows indicate treatment with radiation therapy (3 Gy) performed on two consecutive days. ‡ depicts the time of death due to tumor burden of the indicated mouse. **f** Kaplan–Meier curves showing median survival in BIR and BIR + IR (Ionizing Radiation) groups. (*P* values, two-sided log-rank test). *p < 0.05; **p < 0.001; Student *t*-test

SM bind and neutralize XIAP and target cIAP1 and cIAP2 for proteasomal degradation, leading to cell death via autocrine and paracrine TNFα signaling^10^. Treatment of GSCs with Birinapant (BIR) effectively decreased the expression level of cIAP1 overtime (up to 48 h), while the expression levels of cIAP2 were not significantly affected (Fig. 1a). The SM family of compounds have been previously reported to promote differentiation in GSCs through activation of NF-κB^13;^ however, under our experimental conditions, GSCs treated with BIR failed to reproduce the typical properties observed in differentiated GSCs including reduced cell proliferation, cell death, and increased responsiveness to therapeutic insults. Treatment of GSCs with BIR did not significantly impact cell viability (Fig. 1b). These results are in line with previously reported findings that, given the redundant functions of cIAP1 and cIAP2, downregulation of both IAPs is necessary to sensitize tumors to TNFα-induced cell death^14^. Surprisingly, long-term treatment with a low dose of BIR (once every 3 days over 11 days) increased neurospheres formation and neurospheres size (Fig. 1c), suggesting that SM increase GSCs self-renewal properties. Typically, differentiated GSCs do not form tumors in the brain^15^. To test if long-term treatment with BIR ex vivo affects the tumor initiation properties of GSCs, BT07 GSCs stably expressing Firefly luciferase (Fluc) were treated with 2 µM of BIR for 20 days before orthotopic implantation into the brain of nude mice. Tumor growth and overall survival of mice bearing BIR-treated GSCs did not show any significant differences over the control group (Fig. 1d). These results suggest that BIR treatment fails to induce terminal differentiation and loss of tumor initiating properties in GSCs. SM were reported to sensitize GBM to radiation therapy in vitro^16^. We tested if long-term treatment with BIR at 2 µM enhances the sensitivity of GSCs to radiation therapy. Mice bearing BIR-treated MGG6-Fluc GSCs were treated with either ionizing radiation (IR; 2 consecutive doses of 3 Gy) or control. Unexpectedly, Fluc imaging showed that tumor growth was actually increased following radiation (Fig. 1e) and consequently no survival benefit in the irradiated cohort as compared to the control group was observed (92.5 days for the BIR vs 87.5 days for BIR + IR; Fig. 1f). Overall, these results suggest that GSCs are resistant to cell death induced by BIR and that treatment with this SM stimulated a resistance mechanism in these cells.

### SM induce a prolonged NF-κB activation mediated by TNFα and IL-6

**Fig. 2:**
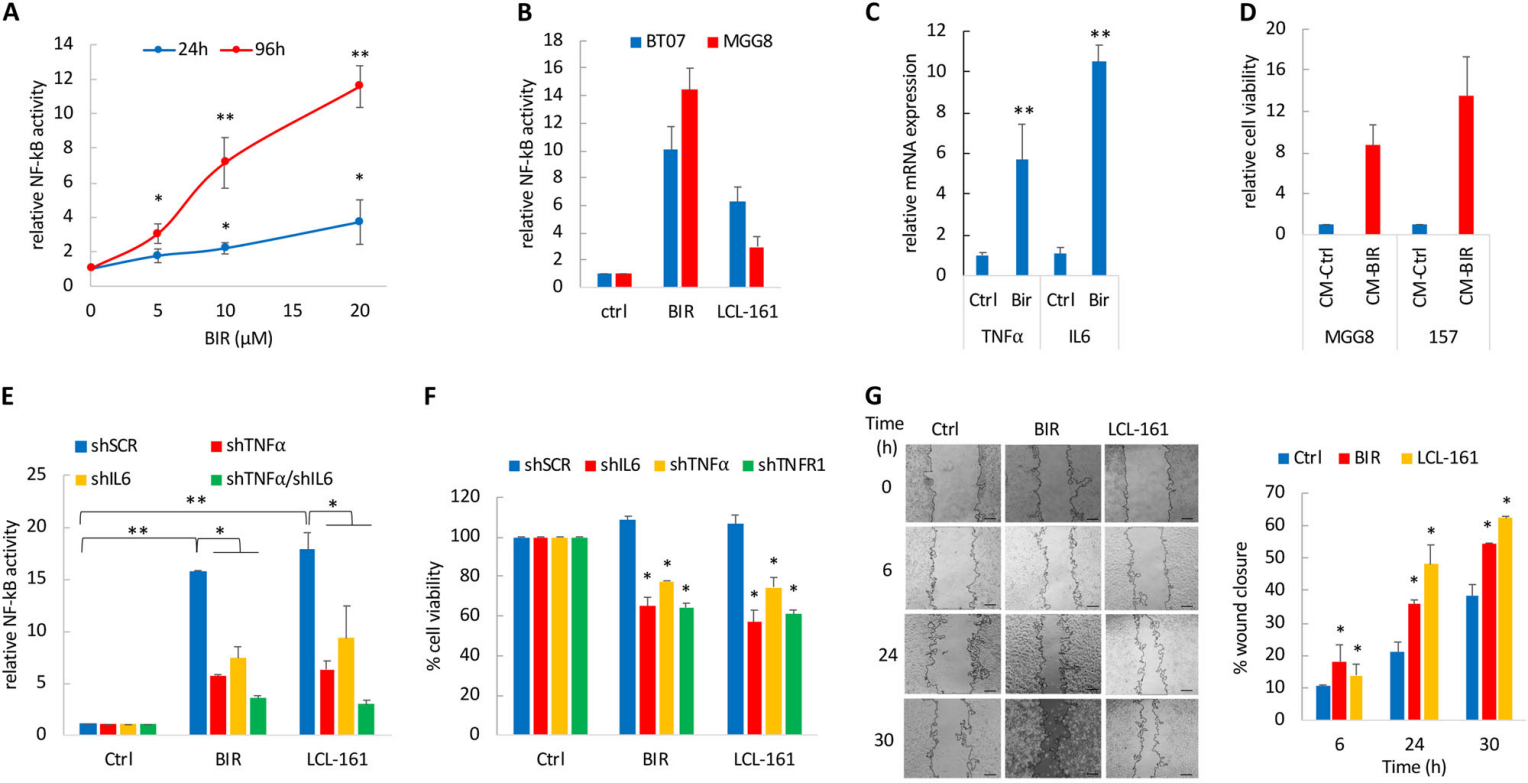
Sustained NF-κB activation following treatment of GSCs with SM. **a** Dose and time-dependent NF-κB activity in MGG8 GSCs expressing an NF-κB reporter and treated with BIR. Data are expressed as fold change of normalized luciferase activity as compared to the untreated control group. **b **Analysis of NF-κB activation in BT07 and MGG8 GSCs at 72 h after treatment with BIR or LCL-161 (10 µM). **c** Fold change in mRNA expression of TNFα and IL-6 normalized to HPRT in GSCs treated with BIR (10 µM) for 6 days, determined by qRT-PCR. **d** MGG8 and 157 GSCs were treated with solvent control or BIR for 4 days after which the conditioned medium (CM) was collected and added on untreated GSCs. Cell viability was analyzed three days later. **e** MGG23 cells expressing the NF-κB reporter were transduced with shSCR, shTNFα, shIL-6 or their combination, treated with BIR or LCL-161 (10 µM) and NF-κB activity was evaluated after 48 h. **f** Cell viability of MGG23 GSCs transduced with shSCR (control), shTNFα, shIL-6 or shTNFR1 and treated with BIR or LCL-161 (10 µM) for 3 days. **g** Scratch wound healing assay in MGG8 cells treated with BIR or LCL-161 (10 µM). Quantification of gap closure was performed overtime and represented as percentage of wound closure compared to time zero. Scale bar, 100 µm. *p < 0.05; **p < 0.001; Student *t*-test

**Fig. 3:**
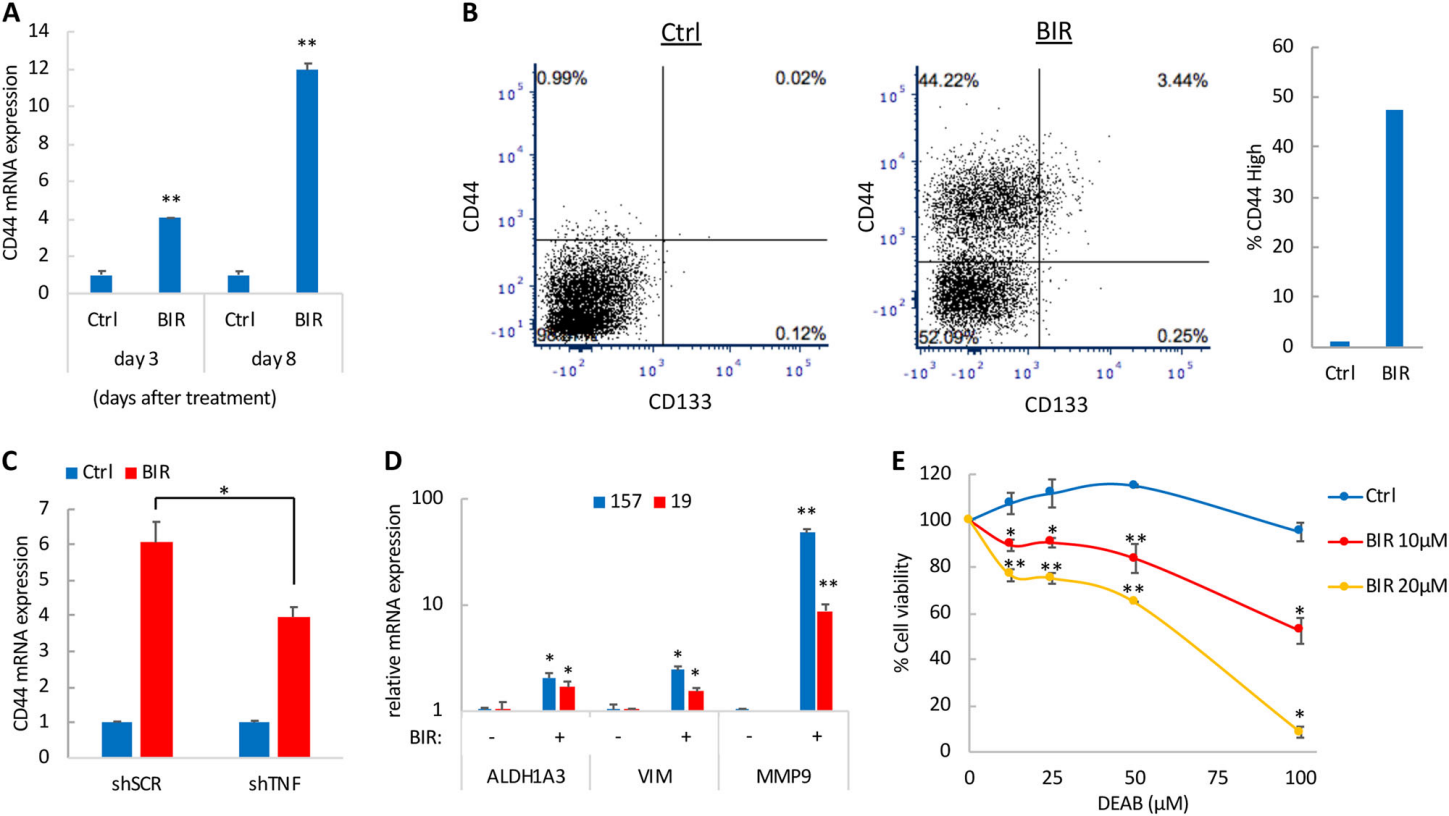
SM promote a mesenchymal transition in GSCs. **a** Relative mRNA expression of CD44 in 157 GSCs treated with BIR (10 µM) at 3 and 8 days, as determined by qRT-PCR. **b** MGG23 GSCs were treated with BIR (10 µM) for 7 days, then analyzed for CD44 and CD133 expression by flow cytometry. The bar graph depicts the percentage of CD44 high cells in Ctrl and BIR-treated GSCs. A representative result from three independent experiment is shown. **c** Relative CD44 mRNA expression in 157 GSCs expressing shSCR or shTNFα and treated with BIR (10 µM) for 7 days. **d** Relative mRNA expression of ALDH1A3, Vimentin, and MMP9 in 157 and 19 GSCs treated with BIR (20 µM) for 4 days. **e** Cell viability of MGG8 GSCs treated with BIR in the presence or absence of DEAB at the indicated doses for 3 days. *p < 0.05; **p < 0.001; Student *t*-test

IAP inhibitors including SM increase the expression of TNFα, a process directly regulated by NF-κB activation^10,17^. We measured NF-κB activity using an NF-κB reporter driving a secreted luciferase^18^, at two different time points following BIR treatment and observed a dose-dependent increase in NF-κB activity following treatment of GSCs with BIR (Fig. 2a). NF-κB activity was further increased at day 4 post-treatment suggesting a sustained and prolonged activation of this transcription factor. Similarly, the SM LCL-161 also induced NF-κB activation in two GSCs (Fig. 2b). Treatment with an IkB kinases (IKK) antagonist, TPCA-1, effectively suppressed NF-κB activation by BIR (Fig. S1a). Long-term treatment with BIR (6 days) also increased the mRNA expression of TNFα and IL-6 (Fig. 2c). Secretion of cytokines such as TNFα is likely to create an autocrine and paracrine stimulation leading to a constitutive activation of transcription factors such as NF-κB. To evaluate the SM-induced paracrine activity, we exposed GSCs to conditioned medium (CM) from GSCs treated with BIR (or vehicle control) and observed a strong increase in cell viability (>8.7-fold increase) as compared to the control group (Fig. 2d). Similarly, CM from GSCs treated with BIR also increased neurospheres formation (Fig. S1b). These results indicate that BIR-induced secretome can promote GSCs proliferation and self-renewal properties. Short hairpin RNA (shRNA)-mediated silencing of TNFα or IL-6 significantly reduced NF-κB activation following BIR treatment (Fig. 2e; Fig. S1c). Additionally, downregulation of TNFα, IL-6, or TNF receptor 1 (TNF-R1) sensitized GSCs to BIR and LCL-161 treatment (Fig. 3f). TNFα promotes cell invasion^19.^ As such, treatment with BIR or LCL-161 significantly increased GSCs migration overtime, as determined by a scratch wound healing assay (Fig. 3g).

### NF-κB activation promotes mesenchymal transition in GSCs treated with SM

NF-κB plays a pivotal role in mesenchymal transition and acquired tumor resistance in gliomas by directly activating regulators of mesenchymal transition such as CD44^20^. A four-fold increase in CD44 mRNA expression was observed 3 days following treatment with BIR, which was further increased to 12-fold, 8 days later (Fig. 3a), in line with the observation that SM induce a sustained NF-κB activation. To corroborate these findings, we analyzed the expression of GSCs putative cell surface markers, CD44 and CD133^21^, upon BIR treatment. We did not observe any changes in CD133 expression, further confirming that SM do not cause differentiation in GSCs; On the other hand, CD44^high^ population was significantly enriched in GSCs treated with BIR (Fig. 3b), likely attributed to TNFα, since this cytokine has been previously shown to potently increase CD44 expression in GSCs^2,22^. Indeed, silencing of TNFα significantly reduced CD44 upregulation after BIR treatment (Fig. 3c). In agreement with the increased NF-κB and CD44 activity, treatment of two GSCs with BIR led to a significant increase in mRNA expression of the mesenchymal markers ALDH1A3 and Vimentin, as well as Matrix metallopeptidase 9 (MMP9), known to promote cancer cell migration and metastasis^23^ (Fig. 3d).

Aldehyde dehydrogenases (ALDH) are upregulated in cancer stem cells. ALDH1A1 and ALDH1A3 have been particularly reported as markers for GSCs^24,25^, with the latter being associated with the mesenchymal subtype^24^. Mesenchymal GSCs present an increased vulnerability to genetic downregulation of ALDH1A3 or pharmacological inhibition of ALDH with N,N-diethylaminobenzaldehyde (DEAB)^24^. Given that SM induce a mesenchymal transition in GSCs, we tested whether inhibition of ALDH with DEAB could reduce cell growth in GSCs treated with SM. Concomitant treatment with BIR or LCL-161 and DEAB at subtoxic doses, resulted in a marked decrease in GSCs viability and a strong synergistic effect (Fig. 3e and S2).

### Treatment of GSCs with SM promotes a TNFα-induced STAT3 activation

**Fig. 4:**
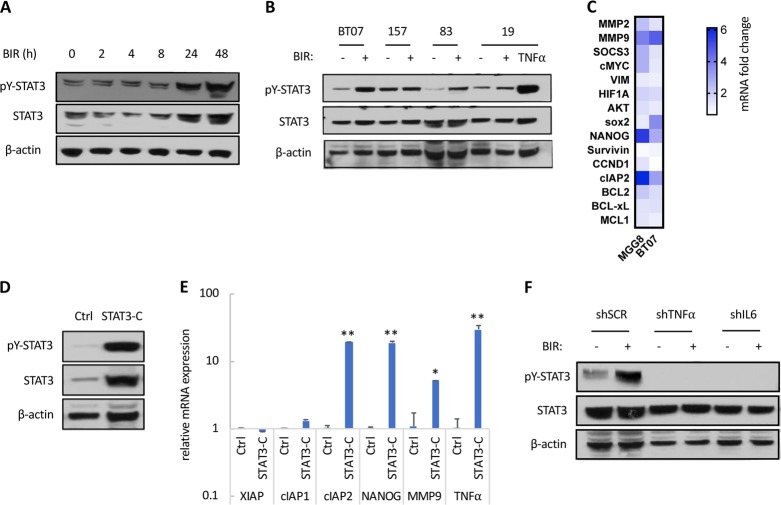
Activation of STAT3 signaling following treatment with SM. **a** Western blot analysis of pY-STAT3 and STAT3 in MGG23 treated with BIR overtime. **b** Western blot analysis of pY-STAT3 and STAT3 in four GSCs treated with BIR or TNFα for 48 h. **c** mRNA expression of STAT3 and NF-κB transcriptional targets in MGG8 and BT07 GSCs treated with BIR for 48 h. Data are normalized to HPRT and expressed as fold change compared to the untreated control. **d** The expression of a constitutively active STAT3-C in GSCs leads to increased pY-STAT3 and STAT3 levels as determined by western blot. **e** Relative mRNA expression of the indicated STAT3 and NF-κB transcriptional targets in MGG8 GSCs expressing a control lentivirus (Ctrl) or STAT3-C. **f** Western blot analysis of pY-STAT3 and STAT3 in 83 GSCs expressing shSCR, shTNFα or shIL-6 and treated with BIR for 48 h

Since TNFα and particularly IL-6 are potent activators of STAT3, we tested whether treatment with SM activates STAT3 signaling in GSCs. We observed a time-dependent increase in STAT3 phosphorylation at tyrosine 705 (pY-STAT3) as well as total STAT3 levels (the STAT3 gene itself is activated upon tyrosine phosphorylation of STAT3), relatively late after treatment, (Fig. 4a), which was further confirmed in three of the four additional GSCs (Fig. 4b). We did not observe any increase in pY-STAT3 in 157 GSCs, which might be attributed to high endogenous levels of activated STAT3 in these cells as compared to the three other GSC specimens (Fig. 4b). These results suggested that STAT3 is indirectly activated by SM, possibly through TNFα and/or IL-6. As expected, treatment with TNFα strongly induced STAT3 phosphorylation (Fig. 4b). To confirm the activation of STAT3 signaling, we analyzed the mRNA expression of validated STAT3 transcriptional targets, which were curated based on their relevant role in GBM and GSCs. Of note, some of these genes such as MMP9 and cIAP2 are common transcriptional targets for both NF-κB as well as STAT3. Several genes, including the pluripotency factor NANOG as well as cIAP2, were upregulated after BIR treatment in two GSCs (Fig. 4c).

Ectopic expression of a constitutively active STAT3 (STAT3-C) in GSCs, which increased pY-STAT3 levels (Fig. 4d), also led to a strong upregulation of NANOG, cIAP2 as well as TNFα transcripts (Fig. 4e), further confirming a role of STAT3 in NANOG and cIAP2 upregulation (the expression levels of XIAP and cIAP1 were unchanged). A strong upregulation of TNFα mRNA expression after constitutive activation of STAT3 (30-fold, Fig. 4e) raised the possibility that a TNFα autocrine signaling is responsible for maintaining a constitutive activation of NF-κB and STAT3 in BIR-treated GSCs. As expected, treatment of GSCs with TNFα activated NF-κB (Fig. S3a) and upregulated TNFα and IL-6 transcripts (Fig. S3b). TNFα also increased the phosphorylation of STAT3 (Fig. 4b) and the expression of MMP9, NANOG, and cIAP2 (Fig. S3b). Additionally, the endogenous levels of pY-STAT3 were decreased following shRNA-mediated downregulation of TNFα or IL-6 (Fig. 4f), confirming previous findings that TNFα activates NF-κB, which in turn induces IL-6, resulting in the activation of STAT3 signaling in gliomas^26^. Finally, silencing of TNFα or IL-6 completely impaired SM-activated STAT3 signaling (Fig. 4f). In summary, treatment of GSCs with SM results in TNFα-mediated activation of STAT3 signaling.

### TNFα promotes the transcriptional activation of cIAP2 following SM treatment

**Fig. 5:**
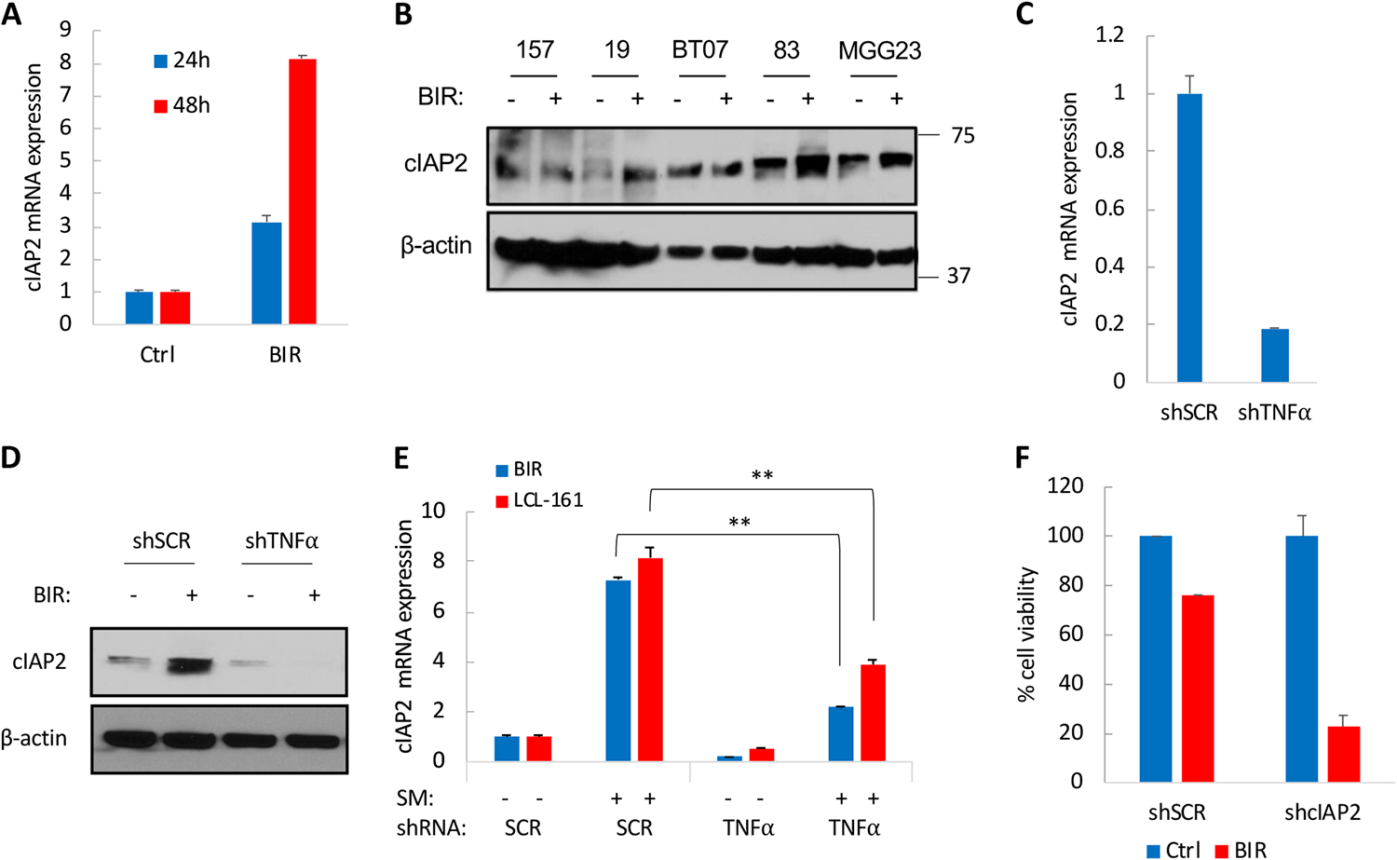
cIAP2 upregulation in GSCs treated with SM. **a** mRNA expression of cIAP2 in GSCs treated with BIR (10 µM) for 24 and 48 h. **b** Protein expression of cIAP2 in five different GSCs after BIR treatment for 72 h. **c** Relative mRNA expression of cIAP2 in GSCs expressing shSCR or shTNFα. **d** Western blot analysis of cIAP2 in GSCs expressing shSCR or shTNFα. **e** cIAP2 levels in GSCs expressing shSCR or shTNFα following treatment with BIR or LCL-161 (10 µM) for 48 h. **f** Cell viability in GSCs expressing shSCR or shcIAP2 and treated with BIR (40 µM) for four days. *p < 0.05; **p < 0.001; Student *t*-test

The observed increase in mRNA expression of cIAP2 after SM treatment was particularly relevant since these compounds target IAPs for degradation and cIAP2 upregulation has been previously reported as a resistance mechanism to SM^14^. The activation of NF-κB by TNFα upregulates IAPs including cIAP2^27^ and indeed treatment of GSCs with TNFα resulted in a 75-fold increase in cIAP2 mRNA expression (Fig. S3b). Notably, whereas treatment of GSCs with BIR for up to 48 h failed to reduce cIAP2 protein levels as initially shown in Fig. 1a, it resulted in an overtime upregulation of cIAP2 transcripts (three-fold at 24 h and up to eight-fold at 48 h post-BIR treatment; Fig. 5a), in line with the aforementioned amplification in NF-κB signaling after SM treatment. These results suggest that increased NF-κB activity overtime is also likely to impact cIAP2 mRNA and protein expression. We therefore assessed cIAP2 protein expression 3 days after treatment with BIR and observed an upregulation of cIAP2 in four out of the five GSCs tested (Fig. 5b). Interestingly, the non-responsive GSC specimen in this experiment was 157 GSC, which did not show an increased phosphorylation of STAT3 after BIR treatment (Fig. 4b), further reinforcing the idea that prolonged STAT3 activation is necessary to maintain cIAP2 expression. To determine whether cIAP2 is a direct transcriptional target of NF-κB or STAT3, we used the STAT3-null prostate cancer cell line PC3. Treatment of the PC3 cell line with BIR resulted in a seven-fold increase in cIAP2 mRNA (Fig. S4a), indicating that cIAP2 is primarily activated by NF-κB. We observed a decrease in cIAP2 mRNA expression (Fig. 5c) as well as cIAP2 protein expression (Fig. 5d) following TNFα knockdown in GSCs. Upregulation of cIAP2 mRNA and protein expression after treatment of GSCs with BIR was significantly impaired following this knockdown (Fig. 5d-e), confirming that TNFα-driven NF-κB activation promotes cIAP2 expression. Similar results were observed with LCL-161 (Fig. 5f), confirming a general mechanism of TNFα-driven NF-κB activation that promotes c-IAP2 expression after treatment of GSCs with SM. Silencing cIAP2 with shRNA decreased GSCs viability after treatment with BIR or LCL-161 (Fig. 5f and Fig. S5b), supporting a role of cIAP2 upregulation in therapeutic resistance to SM. These findings strongly suggest that TNFα-driven upregulation of cIAP2 contributes to the therapeutic resistance.

### Pharmacological targeting of EZH2 sensitizes GSCs to SM-induced cell death

STAT3 could be activated through phosphorylation of Y705 by Janus Associated Kinase 2 (JAK2). We therefore tested the potential role of JAK2 in SM-mediated activation of STAT3. Treatment of GSCs with a JAK2 inhibitor AZD1480 decreased STAT3 activity as evident by a decrease in endogenous pY-STAT3 but failed to completely prevent STAT3 activation after treatment with BIR (Fig. S5a). This JAK2 inhibitor also decreased endogenous and BIR-induced NF-κB reporter activity (Fig. S5b) but did not affect cIAP2 expression (Fig. S5a). We then tested if blocking STAT3 activation (using AZD1480) could sensitize GSCs to SM. The concomitant treatment of AZD1480 with BIR or LCL-161 resulted in a modest decrease in cell viability. Overall, these results suggest an alternative or additional mechanism of activation of STAT3 by SM, independent of JAK2.

**Fig. 6:**
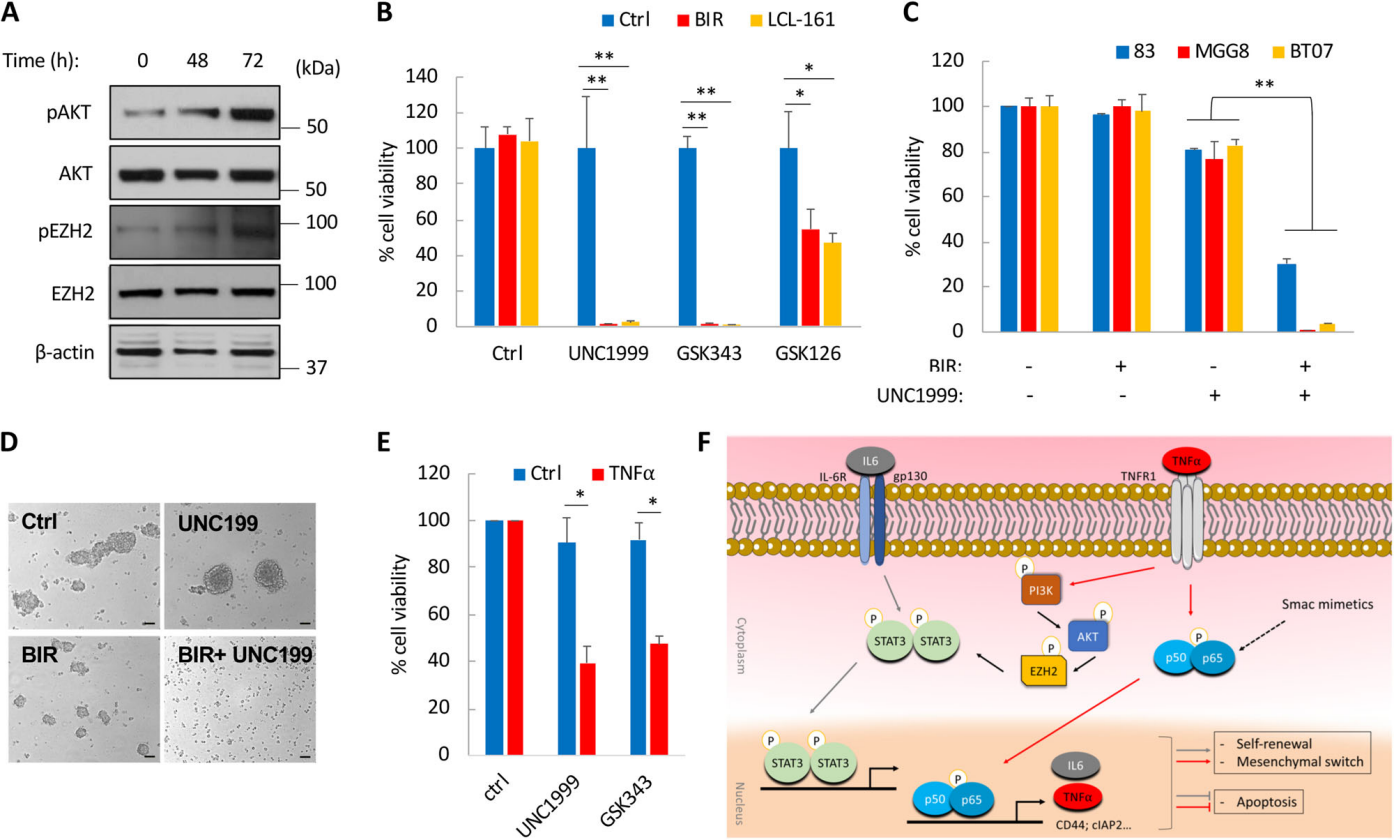
EZH2 inhibition impairs the viability of SM-treated GSCs. **a** Western blot analysis of pAKT (Ser473) and pEZH2 (S21) following treatment with BIR (20 µM) for 0, 48, and 72 h. **b** Cell viability of BT07 GSCs 4 days post-treatment with BIR or LCL-161 (20 µM) in combination with three different EZH2 inhibitors: UNC1999, GSK343, or GSK126. **c** Representative images of BT07 GSCs after treatment with BIR (20 µM) and/or UNC1999 (5 µM) for 3 days. Scale bar, 100 μm. **d** Cell viability of GSCs treated with BIR (20 µM) and UNC1999 (5 µM) for 4 days. **e** Cell viability of GSCs treated with UNC1999 or GSK343 and TNFα (10 ng/ml) for 48 h. *p < 0.05; **p < 0.001; Student *t*-test. **f** Schematic representation of the activated signaling cascades following treatment of GSCs with SM; NF-kB activation promotes an autocrine/paracrine TNFα/IL-6 signaling which in turn activates AKT/EZH2 and STAT3, consequently promoting self-renewal and therapeutic resistance

TNFα activates phosphoinositide-3 kinase (PI-3K) and its downstream target AKT^28^ and promotes GBM proliferation through AKT phosphorylation and activation^29^. Blocking AKT activation with a specific inhibitor of PI-3K/AKT was shown to synergize with the combined treatment with SM and TNFα^14^. In GSCs, AKT mediates the phosphorylation of serine residue 21 of enhancer of zeste homolog 2 (EZH2)^30^. This in turn promotes the interaction between EZH2 and STAT3, which leads to STAT3 methylation and activation^31^. Further, EZH2 is also a regulator of IL-6-dependent demethylation of K49 of STAT3 and essential for the activation of STAT3-mediated transcriptional activation by IL-6^32^. We therefore asked whether treatment of GSCs with SM could activate AKT-EZH2-STAT3 signaling, thus favoring GSCs maintenance and survival. In agreement with a late STAT3 activation in GSCs treated with BIR, we also observed a time-dependent increase in pAKT (Ser473) after 48 and 72 h of treatment with BIR (Fig. 6a). Similarly, we observed an increase in pEZH2 (S21) levels (Fig. 6a and Fig. S5d). We did not detect any significant change in overall expression of EZH2 or its downstream target Histone 3 trimethylated at lysine 27 (H3K27me3) after BIR treatment (Fig. 6a and Fig. S5e). We treated GSCs with BIR or LCL-161 in combination with three selective inhibitors GSK126, GSK343, and UNC1999, which inhibit the catalytic SET domain of EZH2. These inhibitors were used at subtoxic doses, which resulted in little or no cytotoxicity on their own. Combination therapy resulted in a remarkable decrease in GSCs viability particularly with UNC1999 and GSK343 which killed > 90% of GSCs when combined with either BIR or LCL-161 (Fig. 6b). The combination of BIR with UNC1999 also induced loss of neurosphere formation (Fig. 6c), and a strong therapeutic effect in all three GSCs tested (Fig. 6d). Given that TNFα is primarily responsible for NF-κB and STAT3 activation following treatment of GSCs with SM, we reasoned that inhibition of EZH2 could similarly increase GSCs sensitivity to TNFα-induced cell death. Indeed, concomitant treatment of GSCs with TNFα and UNC1999 or GSK343 resulted in >50% decrease in cell viability (Fig. 6e). In summary, we show that EZH2 is essential for GSCs survival after treatment with SM or TNFα, and provide a rationale-based therapeutic combination that effectively depletes GSCs in culture.

## Discussion

Several anti-apoptotic proteins, including IAPs family members such as cIAP1, cIAP2, XIAP, NAIP, and survivin, are upregulated in GSCs^33^. Both cIAP1 and cIAP2 are located on the 11q22 locus, which is frequently amplified in GBM^34^. Further, increased cIAP2 expression was observed in recurrent GBM patients and reportedly induced by temozolomide and radiation therapy suggesting its implication in GBM therapeutic resistance^8^. Therefore, effective targeting of IAPs could serve as a therapeutic strategy to minimize resistance in GBM. We report that the treatment of GSCs with SM promoted defining properties of GSCs including self-renewal, migration, and therapeutic resistance. Key oncogenic signaling such as NF-κB and STAT3, which drive tumor progression and promote a cancer stem cell phenotype in malignancies that include gliomas^26,35^, were activated following SM treatment. This treatment induced a positive and sustained feedback between NF-κB and STAT3, driven by TNFα and IL-6. The molecular signaling cascades activated by SM in GSCs are illustrated in Fig. 6f.

The degradation of cIAPs is critical for SM activation of apoptotic cell death. We report that NF-κB activation in GSCs treated with SM leads to a strong transcriptional activation of cIAP2. Additional regulation mechanisms impacting global cIAP2 protein expression cannot be excluded. For instance, XIAP was reported to stabilize cIAP2 protein levels thus resulting in enhanced IkB-α phosphorylation^36^. On the other hand, decreased levels of cIAP1 protein can increase Receptor-interacting protein 1 (RIP1) binding to TNF receptor 1 (TNF-R1), which in turn results in the activation of NF-κB^17^. Indeed, SM-mediated autocrine production of TNFα was shown to be dependent on RIP1 activity^37^. Given that RIP1 is an essential key switch between prosurvival or a cell death response induced by TNFa^38^, a potential implication of RIP1 in the response of GSCs to SM should be further examined.

The SM BV6 was recently reported to activate NF-κB, thereby promoting astrocytic differentiation of GSCs with loss of tumor initiation properties^39^. These findings are in disagreement with our results whereby BIR and LCL-161 increased self-renewal properties of GSCs by activating NF-κB. Given that NF-κB signaling is often altered and aberrantly active in numerous malignancies including GBM, and its critical role in supporting tumor progression^40^ as well as cancer stem cell maintenance^41^, it is somewhat surprising and unlikely that NF-κB activation would lead to decreased GSCs maintenance. We cannot exclude a superior potency or different mode of action of BV6 as compared to the SM that we tested. It is also possible that an early response to SM is a decrease in GSCs self-renewal and stem cells markers, which is ultimately reversed once NF-κB-STAT3 axis, is activated due to an autocrine/paracrine TNFα signaling. In fact, GSCs have been shown to respond to differentiation stimuli but fail to commit to terminal differentiation^42^. Moreover, GFAP is a member of the cytoskeletal protein family, expressed in astroglial cells and neural stem cells^43^ as well as in astrocytoma and GBM^44,45^. Expression of GFAP is increased following differentiation of GSCs^46,47^, hence it is considered as a surrogate marker for GSCs astrocytic differentiation. The GFAP promoter contains NF-κB binding sites^48,49^, and therefore activation of NF-κB is expected to increase GFAP transcriptional activation without necessarily inducing an astrocytic differentiation in GSCs.

Only a small subset of human cancer cell lines is effectively killed by SM^50^. These targeted agents are likely more effective as adjuvant therapeutics in combination with cytotoxic agents as recently shown in GBM^11,12^. Based on our findings, activation of NF-κB and STAT3 enhances GSCs resistance to SM-induced cell death. However, direct inhibition of NF-κB could lead to undesired side effects caused by immunosuppression and a compromised immune response while specific targeting of STAT3 has been challenging^51^. Therefore, based on mechanistic insight into STAT3 activation in GSCs, we tested an alternative strategy, which combines EZH2 inhibition with SM. This combination therapy resulted in a dramatic decrease of GSCs viability and presents a potent therapeutic strategy. Silencing of EZH2 was reported to decrease XIAP expression in chronic myeloid leukemia cells^52^. As previously discussed, XIAP stabilizes cIAP2 protein levels and therefore could also contribute to the observed SM-induced cytotoxicity following EZH2 inhibition. Increased levels of TNFα in response to SM treatment is a defining feature for such therapeutics and was detected in patients treated with LCL-161 during a phase I study^53^. Therefore, given the observed synergy between SM or TNFα with EZH2 inhibitors such as UNC1999 and GSK343, which are in clinical development, we propose that such combination therapy could be further exploited to target other cancer types.

In conclusion, our study invites caution for therapeutic applications of SM in GBM as a stand-alone therapy. Based on careful evaluation of survival mechanisms following such treatment, we propose a rationale-driven combination therapy with SM to effectively target and deplete GSCs.

## Materials and methods

### Cell cultures

GSCs were derived from surgical specimens obtained from GBM patients at the Massachusetts General Hospital (MGH; provided by Dr. Hiroaki Wakimoto) or The Ohio State University James Comprehensive Cancer Center (provided by Dr. Ichiro Nakano), under the appropriate Institutional Review Board approval and were previously characterized^24,54^. Cells were expanded as neurospheres and maintained in DMEM/F12 supplemented with B27 minus vitamin A (Life Technologies), heparin (Sigma–Aldrich), 20 ng/mL human recombinant EGF, and bFGF-2 (Peprotech). PC3 prostate cancer cell line was obtained from American Type Culture Collection (ATCC) and grown in DMEM with 10% fetal bovine serum. Cells were maintained at 37 °C in humidified 5% CO_2_ incubators.

### Reagents and constructs

Recombinant human TNFα was purchased from Peprotech. The following inhibitors were used in this study: Birnapant, LCL-161, UNC1999, GSK343 (Cayman Chemical), TPCA-1, AZD1480, GSK126 (Selleckchem), DEAB (Sigma–Aldrich). CellTiter-Glo (Promega) was used to measure cell viability. EF.STAT3-C.Ubc.GFP was a gift from Linzhao Cheng (Addgene plasmid # 24983).

### shRNA-mediated gene knockdown

All shRNA used in this study (shTNFα, shIL-6, shTNFR1, shcIAP2, and control shRNA (pLKO.1-puro Non-Target shRNA Control)) were obtained from Sigma (MISSION® shRNA Library) and packaged into lentivirus vectors. GSCs were stably transduced with shRNA lentivirus and selected using puromycin (0.5–1 µg/mL). Knockdown efficiency was determined using qRT-PCR.

### Sphere formation assay

GSCs were dissociated and cells were counted and seeded into a 96-well plate at different cell numbers in eight-replicates. After 7–11 days, neurospheres of >50 μm in diameter were counted.

### Scratch wound healing assay

Cell migration analysis was performed on GSCs plated as an adherent monolayer in the presence of 1 μg/mL Synthemax II-SC substrate (Corning). GSCs were pretreated with SM for 6 h prior to performing scratches. Cells migrating from the leading edge were photographed using phase-contrast microscopy. Distance was measured in a 10 × field using ImageJ (National Institutes of Health).

### Quantitative RT-PCR

Total RNA isolation was performed with RNeasy kit (Qiagen) followed by reverse transcription with OneScript cDNA synthesis Kit (ABM) and real-time PCR using QuantStudio 3 Real-Time PCR system (Applied Biosystems). All primer sequences were obtained from the MGH primer bank and oligonucleotides were synthesized by the CCIB DNA Core Facility at MGH.

### Flow cytometry for CD44 and CD133 expression

Cell surface expression of CD44 and CD133 was analyzed using anti-human CD44-FITC antibody (1:10), anti-human CD133/1-APC (1:10), or immunoglobulin G1 control in the presence of FcR blocking reagent (1:5) (Miltenyi Biotec). GSCs (5 × 10^5^) were dissociated with 2 mM EDTA and incubated in PBS/0.1% bovine serum albumin at 4 °C for 30 min with CD44 and CD133 antibodies. FlTC and APC fluorescence was analyzed using an LSR II Flow Cytometer System (BD Biosciences).

### Immunoblot analysis

Cells were lysed in RIPA buffer (Boston Bio Products) supplemented with 1x protease inhibitors cocktail and 1x PhosSTOP (Roche). Thirty microgram of protein were resolved on 10% NuPAGE Bis-Tris gels (Life Technologies) and transferred to nitrocellulose membranes (Bio-Rad). The following primary antibodies were used in this study: cIAP1 (7065), cIAP2 (3130), Phospho-STAT3 (Tyr705; 9145), STAT3 (4904), Ezh2 (5246), Tri-Methyl-Histone H3 (Lys27; 9733), AKT (4685), Phospho-AKT (Ser473; 9271), and β-Actin (3700) (Cell Signaling Technology); Anti-KMT6/EZH2 (phospho S21; ab84989) (Abcam); sheep anti-mouse IgG-HRP, donkey antirabbit IgG-HRP (Amersham Pharmacia Biotech).

### NF-κB reporter activity

A dual secreted luciferase reporter system was used to monitor NF-κB activity from the conditioned medium as previously described^18^. Cells were transduced with a lentivirus vectors expressing Gaussia luciferase (Gluc) under NF-κB transcription responsive elements, and Vargula luciferase (Vluc) under an SV40 minimal promoter (used as internal control). Aliquots (15 μl) of conditioned medium were collected for luciferase measurement. Coelenterazine (20 µM; Nanolight) and vargulin (5 ng/mL; Nanolight) were the substrates used for detection of Gluc and Vluc activities, respectively, using Synergy HTX multimode reader (Biotek).

### In vivo orthotopic GSCs model

All animal experiments were approved by the MGH Subcommittee on Research Animal Care. GSCs (5 × 10^4^ cells) expressing Firefly luciferase (Fluc) were stereotactically implanted into the left forebrain of nude mice (2.5 mm lateral and 0.5 mm anterior to bregma, at a 2.5 mm depth from the skull surface). Tumor growth was monitored by Fluc bioluminescence imaging using a Xenogen IVIS 200 Imaging System (PerkinElmer), after intraperitoneal (i.p.) injections of D-luciferin (150 mg/kg body weight) (Gold Biotech). Image intensity was quantitated using the Living Image software 4.3.1 (PerkinElmer). Ionizing radiation treatment in mice was performed using a^137^Cs irradiator.

### Statistical analysis

All results are presented as mean ± SD. All measurements were performed using at least three biological replicates. *P* < 0.05 was considered as statistically significant. Student *t*-test was used to measure significance between two datasets. For survival analysis, significance was measured with log-rank test using GraphPad Prism 7.

## Supplementary information

supplementary figure legends

Figure S1

Figure S2

Figure S3

Figure S4

Figure S5
