# Author Correction: Sustained NF-κB-STAT3 signaling promotes resistance to Smac mimetics in Glioma stem-like cells but creates a vulnerability to EZH2 inhibition

**DOI:** 10.1038/s41420-019-0183-5

**Published:** 2019-06-17

**Authors:** Cintia Carla da Hora, Kelsey Pinkham, Litia Carvalho, Max Zinter, Elie Tabet, Ichiro Nakano, Bakhos A. Tannous, Christian E. Badr

**Affiliations:** 1 Department of Neurology, Massachusetts General Hospital, Boston, MA USA; 2 Neuroscience Program, Harvard Medical School, Boston, MA USA; 3 Experimental Therapeutics and Molecular Imaging Laboratory, Charlestown, MA USA; 4 Department of Neurosurgery and Comprehensive Cancer Center, University of Alabama at Birmingham, Birmingham, AL USA

**Keywords:** Cancer stem cells, CNS cancer

**Correction to:** ***Cell Death Discovery***

10.1038/s41420-019-0155-9, published online 04 March 2019

After publication of this article, the authors realized that the Supplementary Figure S5B had an error in it. Specifically, the labels for BIR and AXD1480 on the x-axis were in the wrong place. All text referring to the figure, including the legend, are correct and this does not impact the findings of the study. The corrected figure is supplied below. We apologize to readers for the inconvenience.

**Fig. S5B:**
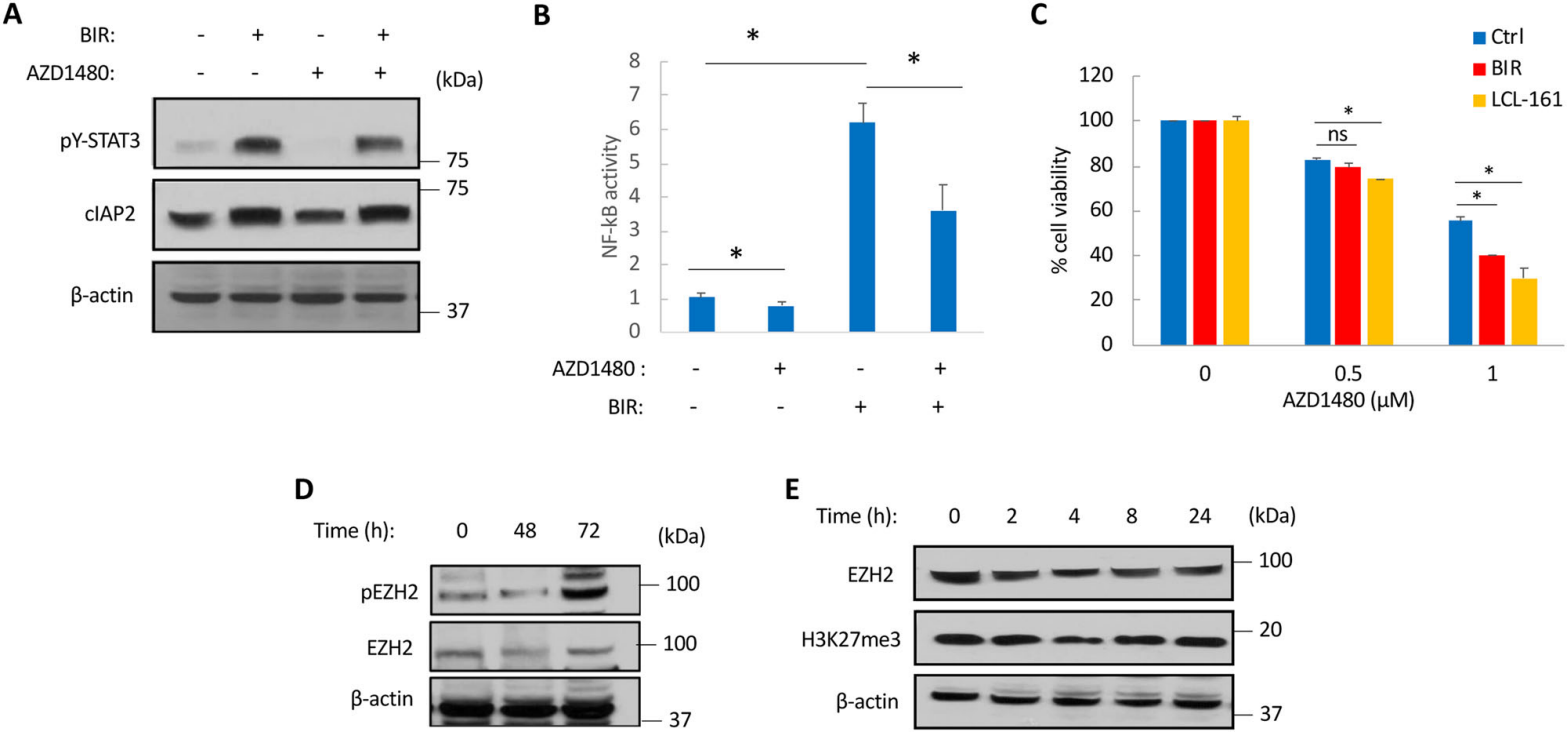
▪

