# Supplementary material for: Sustained NF-κB-STAT3 signaling promotes resistance to Smac mimetics in Glioma stem-like cells but creates a vulnerability to EZH2 inhibition
Source: Cell Death Discov. 2019 Mar 4;5:72. doi: 10.1038/s41420-019-0155-9 (PMC6399311; doi:10.1038/s41420-019-0155-9)
Supplement: Supplementary file 1 — supplementary figure legends [file 41420_2019_155_MOESM1_ESM.pdf]

Figure S1: **A.** MGG8 stably expressing dual NF-κB reporter (where Gluc is under the control of NF-κB response elements and Vluc under SV40 promoter as an internal control) were pretreated with TPCA for 1h followed by BIR (2 μM). NF-κB activity was measured after 24 and 48h of treatment by analyzing Gluc/Vluc levels. **B.** MGG8 were dissociated, counted and plated in the presence of conditioned medium collected from control or BIR-treated GSCs. Spheres were counted 7 days post-treatment. **C.** 157 GSCs expressing the NF-κB reporter were stably transduced with shSCR (control), shTNFα or shIL-6 and treated with BIR or LCL-161 (10 μM). NF-κB activity was analyzed 48h later. ns: not significant; *$p<0.05$; **$p<0.001$; Student t-test.

Figure S2: **A.** MGG8 GSCs were co-treated with the indicated doses of LCL-161 in the presence or absence of DEAB (100 μM) and cell viability was measured two days later. ** $p<0.001$; Student t-test.

Figure S3: **A.** MGG8 GSCs expressing the NF-κB reporter were treated with TNFα (10 ng/ml) and NF-κB activation was analyzed after 24h. **B.** Relative mRNA expression of STAT3 and NF-κB transcriptional targets in GSCs treated with TNFα. *$p<0.05$; **$p<0.001$; Student t-test.

Figure S4: **A.** mRNA expression of cIAP2 in PC3 prostate cancer cells treated with BIR (10 μM) for 24h. **B**. MGG23 GSCs were transduced with shSCR or shcIAP2 and subsequently treated with LCL-161 at the indicated doses. Cell viability was measured three days after treatment. *$p<0.05$; **$p<0.001$; Student t-test.

Figure S5: **A.** Western blot analysis of pY-STAT3 and cIAP2 in 83 GSCs treated with BIR (10

μM) and/or AZD1480 (2 μM). **B.** NF-κB activity following treatment of MGG8 GSCs expressing dual reporter with BIR and AZD1480. **C.** Cell viability in MGG8 GSCs treated with BIR or LCL-161 with the indicated concentrations of AZD1480 for four days. **D.** Western blot analysis of pEZH2 (S21) in MGG8 GSCs following treatment with BIR (20 μM) for 0, 48 and 72h **E.** Western blot analysis of EZH2 and H3K27me after treatment with BIR (10 μM) at different time points. ns: not significant; * $p<0.05$; ** $p<0.001$; Student t-test.
