# Supplementary figures and images for: Sustained NF-κB-STAT3 signaling promotes resistance to Smac mimetics in Glioma stem-like cells but creates a vulnerability to EZH2 inhibition
Source: Cell Death Discov. 2019 Mar 4;5:72. doi: 10.1038/s41420-019-0155-9 (PMC6399311; doi:10.1038/s41420-019-0155-9)

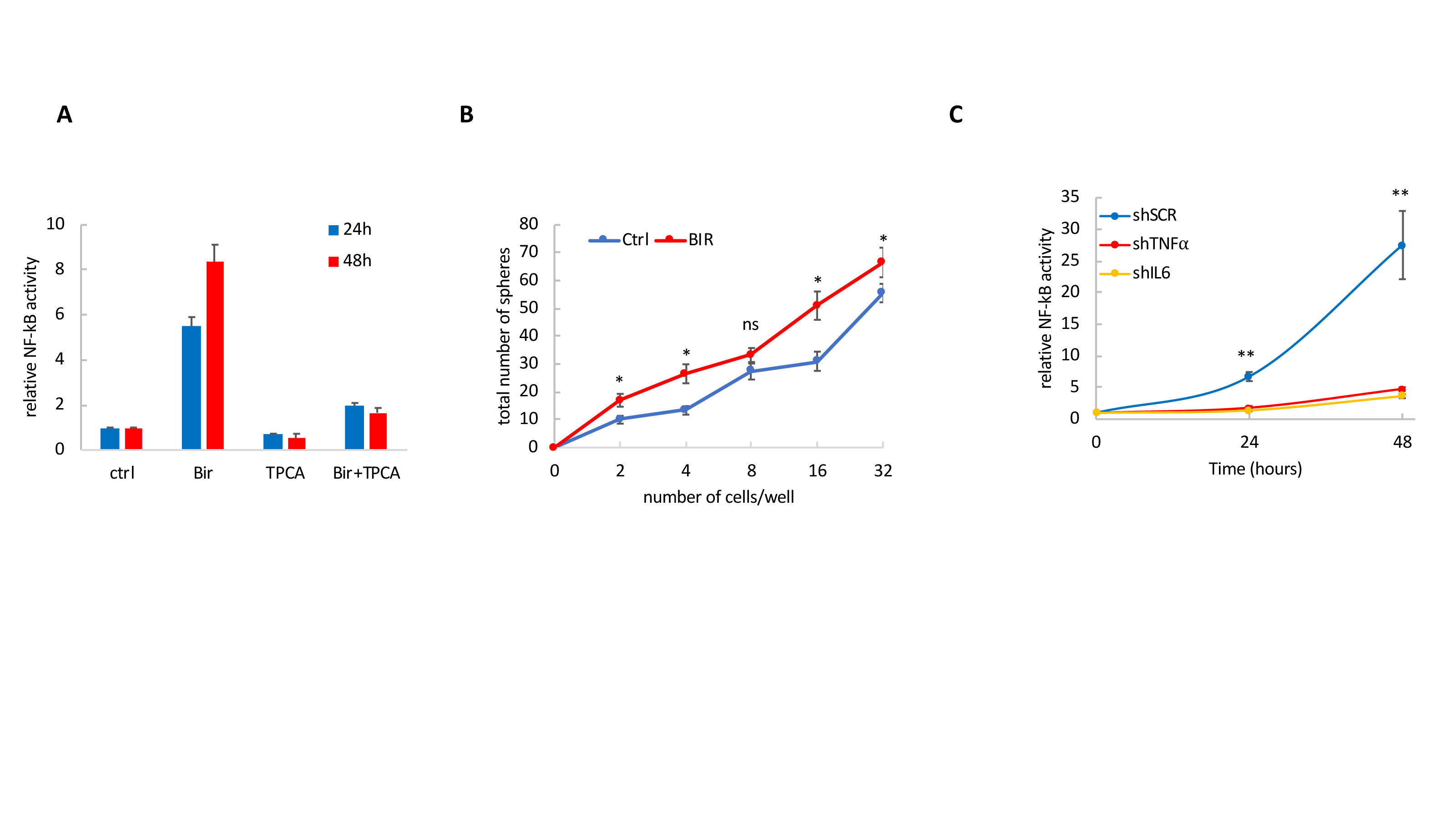

Supplement: Supplementary file 2 — Figure S1 [file 41420_2019_155_MOESM2_ESM.tif]

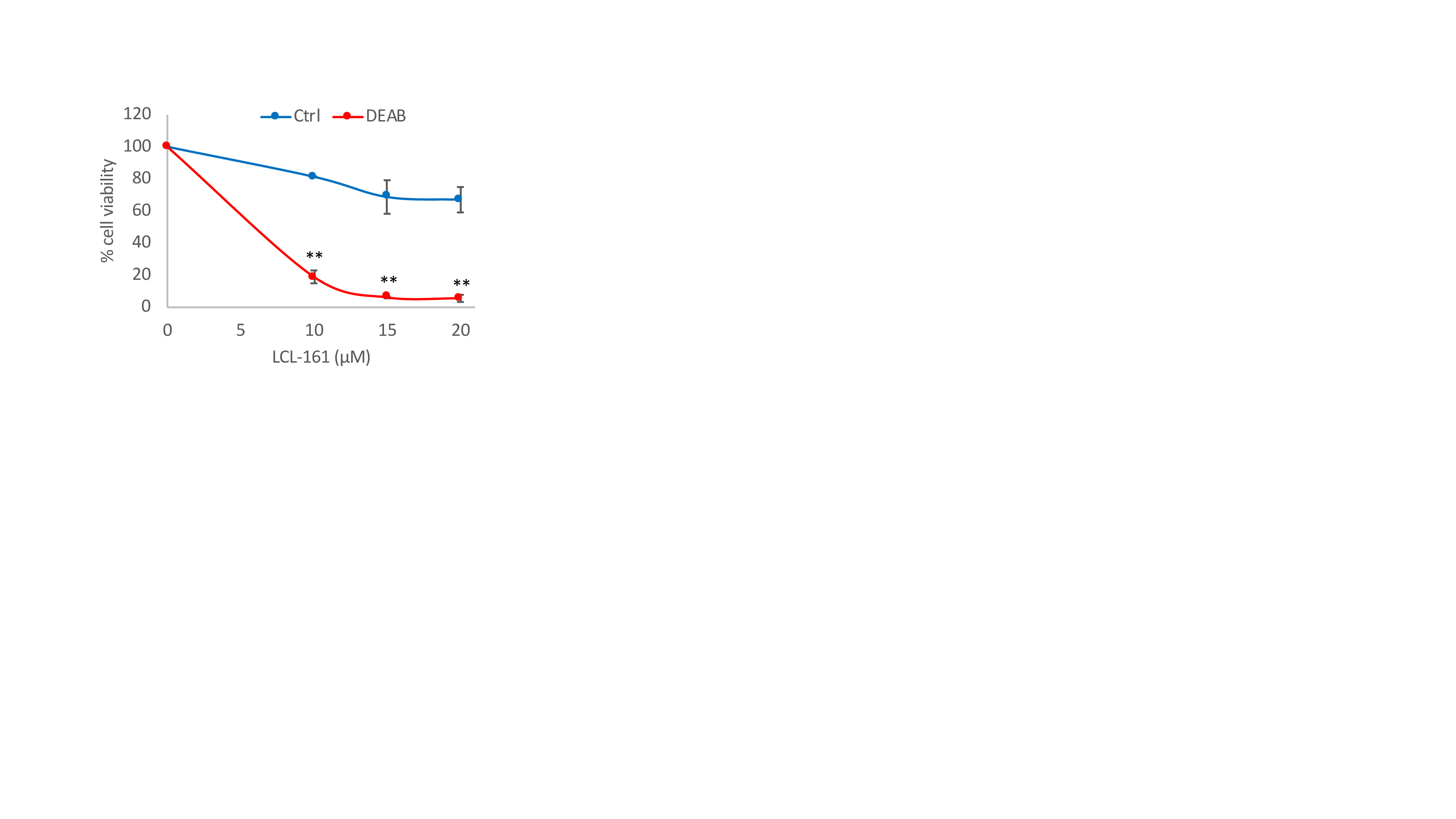

Supplement: Supplementary file 3 — Figure S2 [file 41420_2019_155_MOESM3_ESM.tif]

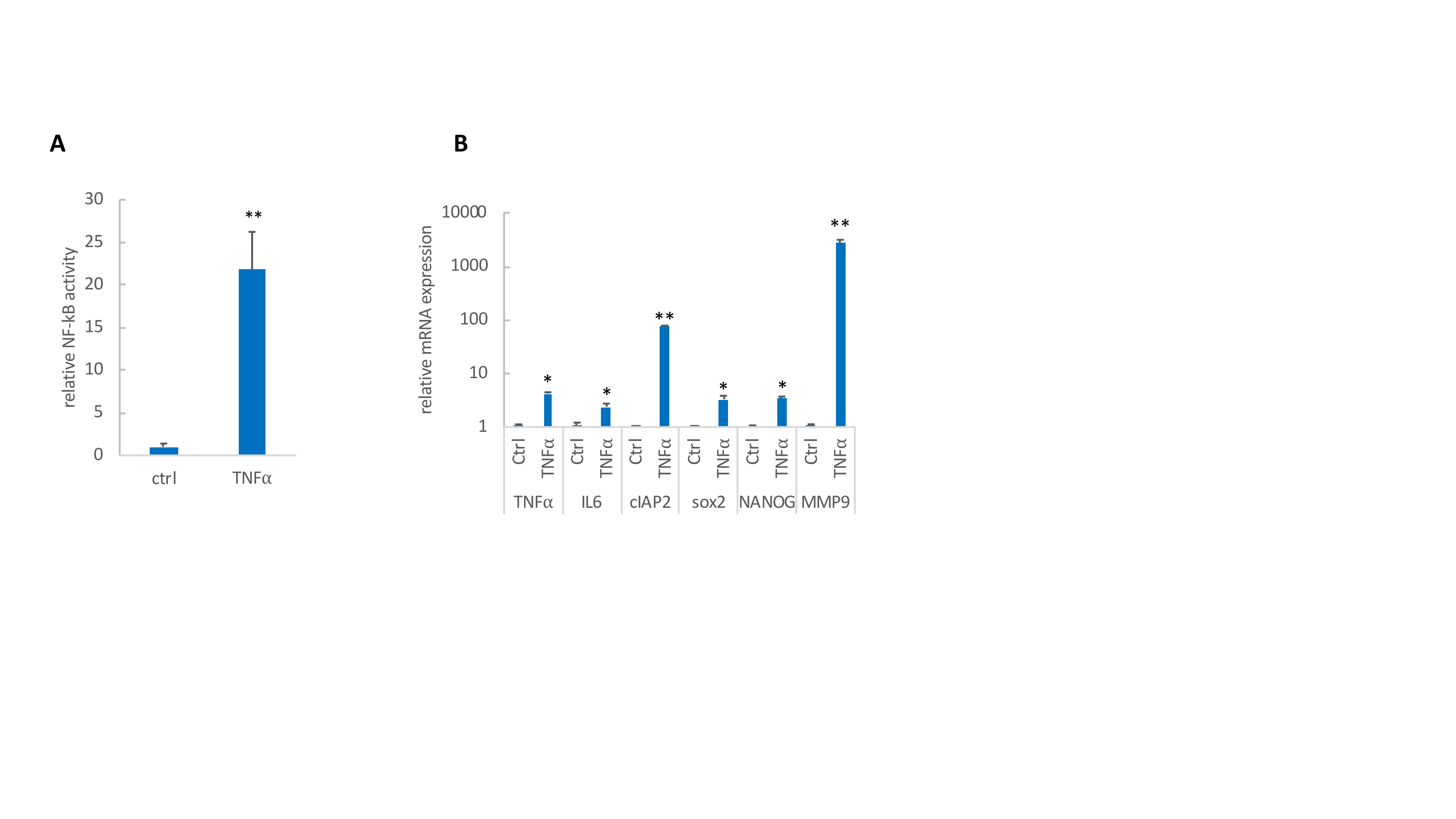

Supplement: Supplementary file 4 — Figure S3 [file 41420_2019_155_MOESM4_ESM.tif]

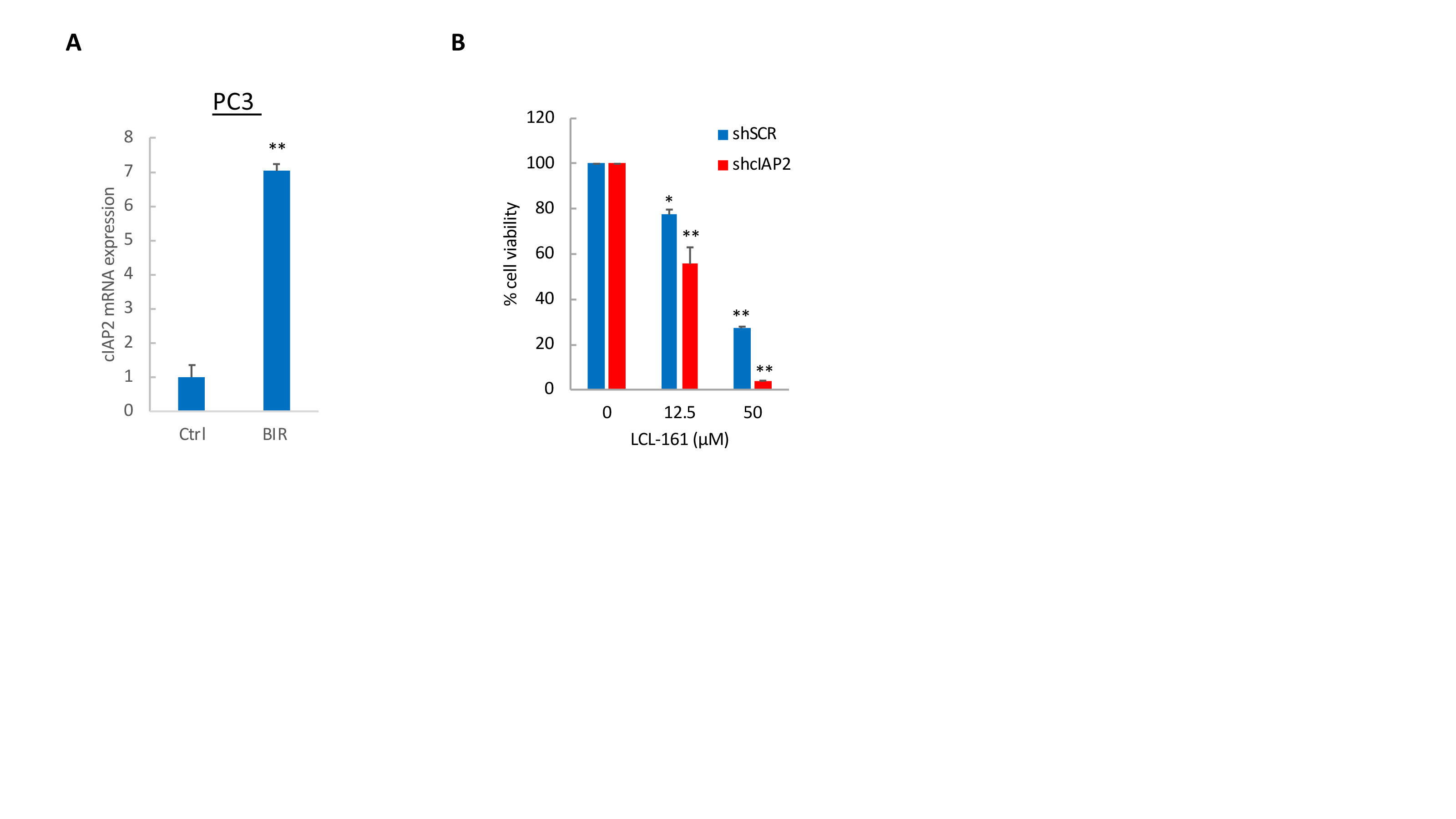

Supplement: Supplementary file 5 — Figure S4 [file 41420_2019_155_MOESM5_ESM.tif]

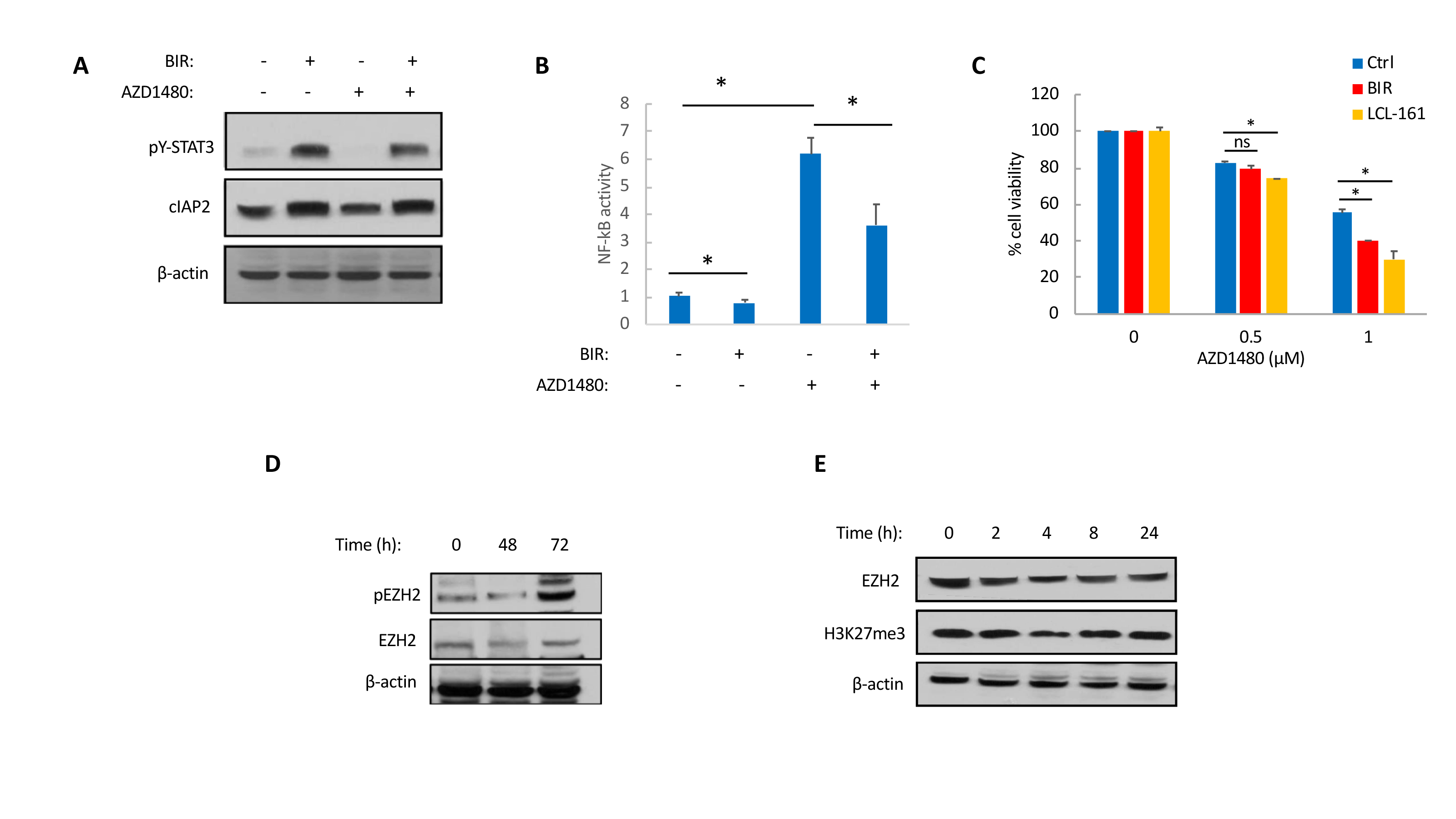

Supplement: Supplementary file 6 — Figure S5 [file 41420_2019_155_MOESM6_ESM.tif]
